# Effect on comfort of administering bubble-humidified or dry oxygen: the Oxyrea non-inferiority randomized study

**DOI:** 10.1186/s13613-018-0472-9

**Published:** 2018-12-17

**Authors:** Laurent Poiroux, Lise Piquilloud, Valérie Seegers, Cyril Le Roy, Karine Colonval, Carole Agasse, Vanessa Zinzoni, Vanessa Hodebert, Alexandre Cambonie, Josselin Saletes, Irma Bourgeon, François Beloncle, Alain Mercat, for the REVA Network

**Affiliations:** 1 Medical Intensive Care Department, Angers University Hospital, 4, rue Larrey, 49933 Angers Cedex, France; 2 Adult Intensive Care and Burn Unit, Medical Intensive Care Department, Lausanne University Hospital, rue du Bugnon 46, 1011 Lausanne, Switzerland; 3 Département de Biométrie, Institut de Cancérologie de l’Ouest, 15 avenue Bocquel, 49055 Angers Cedex 02, France; 4 Medical Intensive Care Department, Orléans Regional Hospital, 4 avenue de l’hôpital, 45067 Orléans Cedex, France; 5 Medical Intensive Care Department, Nantes University Hospital, 1 place Alexis-Ricordeau, 44093 Nantes Cedex 1, France; 6 Intensive Care Department, La Roche-sur-Yon Hospital, Boulevard Stéphane Moreau, 85925 La Roche-Sur-Yon, France; 7 Intensive Care Unit, Saint-Malo Hospital, 1 Rue de la Marne, 35400 Saint-Malo, France; 8 Medical Intensive Care Department, Poitiers University Hospital, 2 rue de la Milétrie, 86000 Poitiers, France; 9 Intensive Care Unit, Le Mans Hospital, 194 avenue Rubillard, 72037 Le Mans Cedex 9, France; 10 Medical Intensive Care Department, Henri Mondor University Hospital, 51 avenue du Maréchal de Lattre de Tassigny, 94010 Créteil, France

**Keywords:** Oxygen therapy, Bubble humidification, Patient comfort, Nursing assessment, Intensive care units

## Abstract

**Background:**

The clinical interest of using bubble humidification of oxygen remains controversial. This study was designed to further explore whether delivering dry oxygen instead of bubble-moistened oxygen had an impact on discomfort of ICU patients.

**Methods:**

This randomized multicenter non-inferiority open trial included patients admitted in intensive care unit and receiving oxygen. Any patient receiving non-humidified oxygen (between 0 and 15 L/min) for less than 2 h could participate in the study. Randomization was stratified based on the flow rate at inclusion (less or more than 4 L/min). Discomfort was assessed 6–8 and 24 h after inclusion using a dedicated 15-item scale (quoted from 0 to 150).

**Results:**

Three hundred and fifty-four ICU patients receiving non-humidified oxygen were randomized either in the humidified (HO) (*n* = 172), using bubble humidifiers, or in the non-humidified (NHO) (*n* = 182) arms. In modified intention-to-treat analysis at H6–H8, the 15-item score was 26.6 ± 19.4 and 29.8 ± 23.4 in the HO and NHO groups, respectively. The absolute difference between scores in both groups was 3.2 [90% CI 0.0; + 6.5] for a non-inferiority margin of 5.3, meaning that the non-inferiority analysis was not conclusive. This was also true for the subgroups of patients receiving either less or more than 4 L/min of oxygen. At H24, using NHO was not inferior compared to HO in the general population and in the subgroup of patients receiving 4 L/min or less of oxygen. However, for patients receiving more than 4 L/min, a post hoc superiority analysis suggested that patients receiving dry oxygen were less comfortable.

**Conclusions:**

Oxygen therapy-related discomfort was low. Dry oxygen could not be demonstrated as non-inferior compared to bubble-moistened oxygen after 6–8 h of oxygen administration. At 24 h, dry oxygen was non-inferior compared to bubble-humidified oxygen for flows below 4 L/min.

**Electronic supplementary material:**

The online version of this article (10.1186/s13613-018-0472-9) contains supplementary material, which is available to authorized users.

## Background

Oxygen administration is commonly recognized as a source of discomfort [1], potentially linked to insufficient moisture of the administered gas [2–4]. One study published by Andres et al. [5] demonstrated that bubble humidification could slightly improve comfort in patients requiring oxygen at flow rates < 4 L/min. The improvement in comfort was, however, too small to be clinically relevant. By contrast, Wells et al. [6] showed that non-heated reservoirs temperature was too low to obtain significant humidity in delivered oxygen. In line, Estey et al. [7] and Campbell et al. [2] demonstrated the inability of bubble humidification to improve comfort, respectively, in patients receiving oxygen at flow rates < 4 and > 5 L/min. More recently, Chanques et al. [3] reported that one-third of the patients receiving bubble-humidified oxygen at flow rates > 5 L/min complained of moderate-to-severe discomfort often due to dry nose and/or throat feeling. This also suggests a poor effect of bubble humidification, at least for high oxygen flows. Finally, a recent meta-analysis [8] underlined the absence of benefice of using bubble humidifiers on nose and throat dryness sensation, nose bleeding, chest discomfort and abnormal smell perceived by the patients. Prospective data are still missing to definitely conclude. Despite the lack of evidence, the 1984 [9], the 2002 [10] and the 2017 [11] guidelines stated that there were no objective evidences that routine humidification of oxygen using bubble humidifiers was useful for flow rates of 1–4 L/min. No recommendations were given for higher flows. Nevertheless, bubble humidifiers are extensively used in daily clinical practice.

Our hypothesis was that delivering dry oxygen to critically ill patients was not inferior than administering bubble-humidified oxygen in terms of induced discomfort. The main aim of the present study was thus to compare discomfort associated with dry and bubble-humidified oxygen administration.

## Methods

### Trial design

This study was a randomized (allocation ratio 1:1) multicenter non-inferiority open trial. It took place from March 2011 to August 2014 in nine university and non-university ICUs in France. The list of the participating centers with the number of inclusions is given in Additional Files (AF) (Table AF-1). This study was approved by the leading hospital ethics committee (Comité de Protection des Personnes Ouest II) under the reference 2010-24. It was performed in accordance with the ethical standards laid down in the 1964 Declaration of Helsinki and its later amendments. According to French laws, as studied oxygen administration strategies are both considered as standard of care, written informed consent was not required. The investigators informed patients about the trial orally and with a written document. Patients were informed that they could decline to participate at any time, and their decision was recorded in patient files. The study was registered in ClinicalTrials.gov (NCT01300845).

### Participants

Any patient hospitalized in the ICU receiving non-humidified oxygen (between 1 and 15 L/min) through nasal cannula or face mask for less than 2 h could participate in the study. Non-inclusion criteria were age < 18 years, pregnancy, protected adults, non-affiliated to social security system patients, tracheostomized patients, oxygen therapy probably required for less than 6 h, patients previously ventilated during the hospital stay, patients unable to respond to the questionnaire, patients who had already participated in the study and patients in palliative treatment.

### Randomization

Patients were randomized in the bubble-humidified oxygen group (HO) or in the non-humidified oxygen group (NHO). As the published recommendations suggest that humidification could be particularly useless at flow rates ≤ 4 L/min [10], randomization was stratified for oxygen flow rates at inclusion lower or higher than this threshold.

### Interventions

In the HO arm, the Aquapak^®^ humidifier (Teleflex, Inc., Morrisville, NC USA) was used and humidification was started as soon as possible after randomization. The humidifier was connected to an oxygen flowmeter and to the oxygen administration interface (nasal cannulas or masks) selected by the clinicians according to their usual practice.

### Outcomes

The primary endpoint was a 15-item score of discomfort assessed between the 6th and 8th hour (H6–H8) after inclusion. This score, quoted from 0 to 150 points (0 meaning no discomfort and 150 worst discomfort), was calculated by adding the points (0–10) given by the patient to each of the fifteen following items: oral dryness, oral burning sensation, speaking difficulty, thirst, throat dryness, sore throat and/or swallowing difficulty, sensation of coolness or warmth in airways, nasal dryness, greater need to blow the nose, particular smell sensation, eye discomfort, chest discomfort, headache, discomfort related to ambient noise and abnormal taste in the mouth. Practically to standardize the oxygen-related discomfort assessment, a health practitioner asked the patient to quote orally each of the fifteen items of the scale one by one by scoring a level of discomfort between 0 and 10. The fifteen items were selected from data reported in the literature at the time of the study beginning [2, 5, 12] and from a previous survey conducted in the University Hospital of Angers ICU in 50 patients receiving oxygen. The patients were asked to mention the main reason of discomfort associated with oxygen therapy. To create the 15-item final score, only criteria mentioned by two patients or more were selected. The 15-item discomfort scale and its validation process are illustrated in AF (figures AF-1 and AF-2). Both the global 15-item score values and the percentages of patients with a discomfort score of more than 45/150 were recorded. Planned secondary endpoints were the global 15-item discomfort scores recorded at 24 h (H24) after inclusion, the number of patients who were intubated during the ICU stay, the number of patients who developed an ear, nose or throat (ENT) infection or a corneal lesion during the ICU stay, the number of patients who required a bronchoscopy, ICU length of stay and ICU mortality. When required, the secondary outcomes were censured at 28 days. As a post hoc analysis, we also considered the percentages of patients with a discomfort score of more than 45/150 at H24.

### Sample size

As the global 15-item discomfort score was used for the first time, the mean expected values and their dispersion were not known. Consequently, no formal a priori sample size calculation could be performed. On the basis of the Campbell study results [2], it was anticipated to record a mean 15-item discomfort score of about 45/150 points with a relatively low dispersion of the values (standard deviation of the mean of less than 20% of the mean value). Thereby, a sample size of 350 patients (175 by group) was anticipated to be large enough to conclude.

### Statistical methods

Only the patients for whom the primary outcome was available were included in the analyses. A non-scored item of the 15-item comfort score was computed as 0. According to the CONSORT recommendation [13], for this non-inferiority trial, the primary endpoint results are presented as the absolute difference (with 90% confidence interval—CI) between the NHO and HO groups. The null hypothesis was defined as “mean comfort score value in the HO group is more than 20% lower than mean comfort score value in the NHO group.” Thus, the non-inferiority margin was computed as the mean comfort score of the HO group + 20%. Non-inferiority is accepted if this margin is not comprised within the CI of the difference between HO and NHO comfort scores and if the 90% CI includes 0 [13]. The primary endpoint analysis and all the comfort score comparisons were performed for non-inferiority in per protocol (PP) and modified intention-to-treat analysis (mITT). mITT means an intention-to-treat analysis after exclusion of the patients who did not meet inclusion criteria or/and who had a posteriori an exclusion criterion. Normality of continuous variables was assessed using the Shapiro–Wilk test. According to the usually performed non-inferiority analyses, means and standard deviations were used to define non-inferiority thresholds, independently from the normality of the data distribution. Statistical analyses were performed with R, Core Team (2018), version 3.3.3 (Vienna, Austria). A *p* value < 0.05 was considered as statistically significant (bilateral tests for all comparisons except for non-inferiority analyses). For quantitative variables, statistical tests used were the Student’s *t* test or the nonparametric Mann–Whitney test as indicated. Comparisons between qualitative variables were made using the Chi-square or Fisher’s exact tests as indicated.

## Results

### Participants

**Fig. 1:**
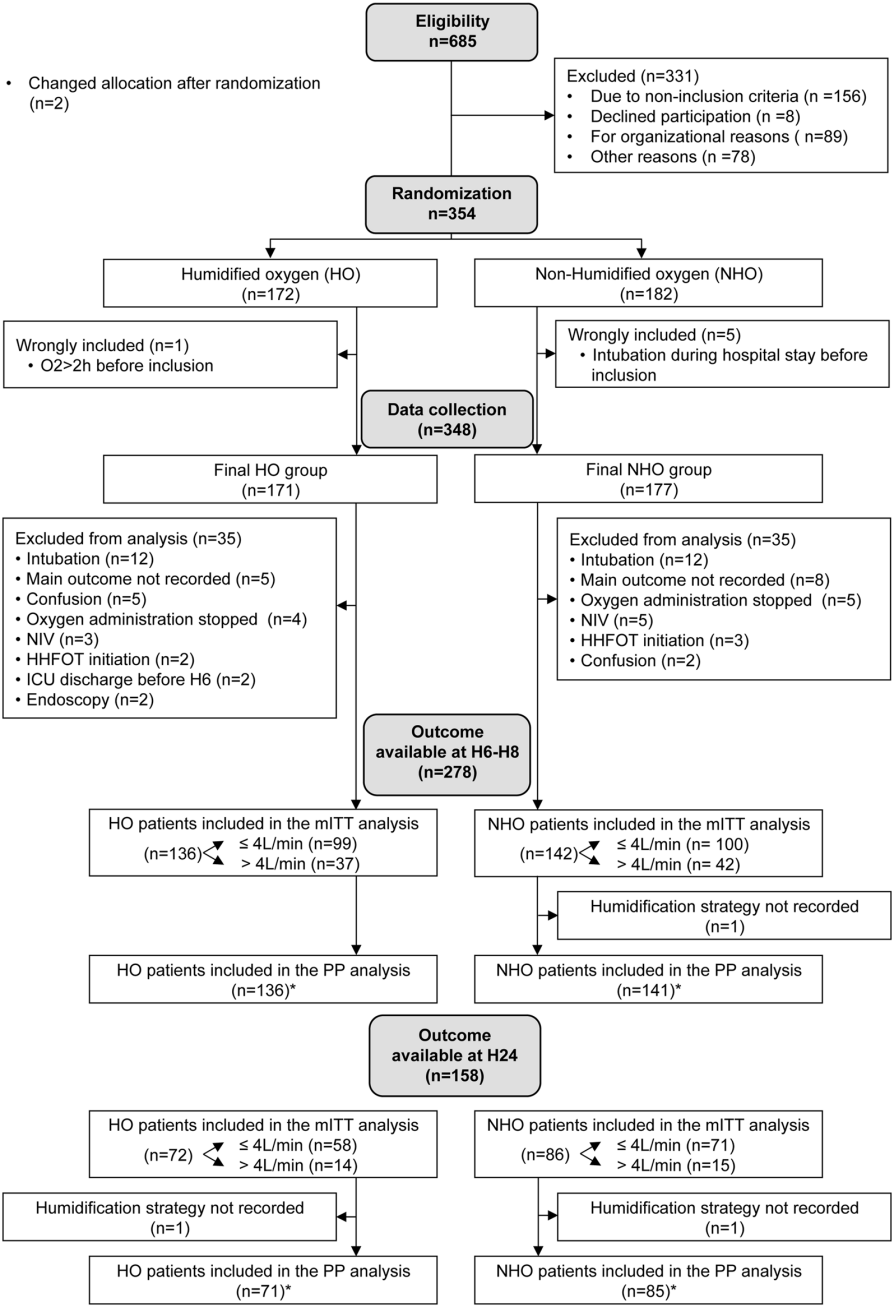
Study flowchart. *NIV* noninvasive ventilation, *HHFOT* humidified high flow oxygen therapy, *ICU* intensive care unit

Three hundred and fifty-four patients were randomized in the HO (*n* = 172) or in the NHO (*n* = 182) arms. Figure 1 illustrates the flowchart of the study. Patients’ characteristics are mentioned in Table 1.

**Table 1:** Patients’ characteristics, reasons for intensive care unit (ICU) admission and past medical history

Characteristics	Humidified oxygen therapy (HO group) (*n* = 136)	Non-humidified oxygen therapy (NHO group) (*n* = 142)
Age (years), median (IQR)	64 (51–77)	63.5 (54.25–74)
Male sex, *n* (%)	94 (69.1%)	91 (64.1%)
Previous use of oxygen in hospital before ICU admission, *n* (%)	90 (66.2%)	103 (72.5%)
Previous use of oxygen at home, *n* (%)	3 (2.2%)	8 (5.7%)
Reasons for ICU admission		
Respiratory failure, *n* (%)	50 (36.8%)	39 (27.5%)
Cardiac failure/lung edema, *n* (%)	2 (1.5%)	12 (8.5%)
Sepsis, *n* (%)	34 (25.0%)	39 (27.5%)
Renal and metabolic failure, *n* (%)	15 (11.0%)	15 (10.6%)
Others, *n* (%)	35 (25.7%)	37 (26.1%)
Illness potentially influencing evaluation of discomfort score		
ENT cancer, *n* (%)	5 (3.7%)	5 (3.5%)
Sjögren’s syndrome, *n* (%)	0 (0%)	1 (0.7%)
Past cervical or facial radiotherapy, *n* (%)	4 (2.9%)	4 (2.8%)
Past or current tobacco use, *n* (%)	67 (49.3%)	64 (45.4%)

*ENT* ear, nose and throat, *HO* humidified oxygen, *NHO* non-humidified oxygen

### Outcomes

**Fig. 2:**
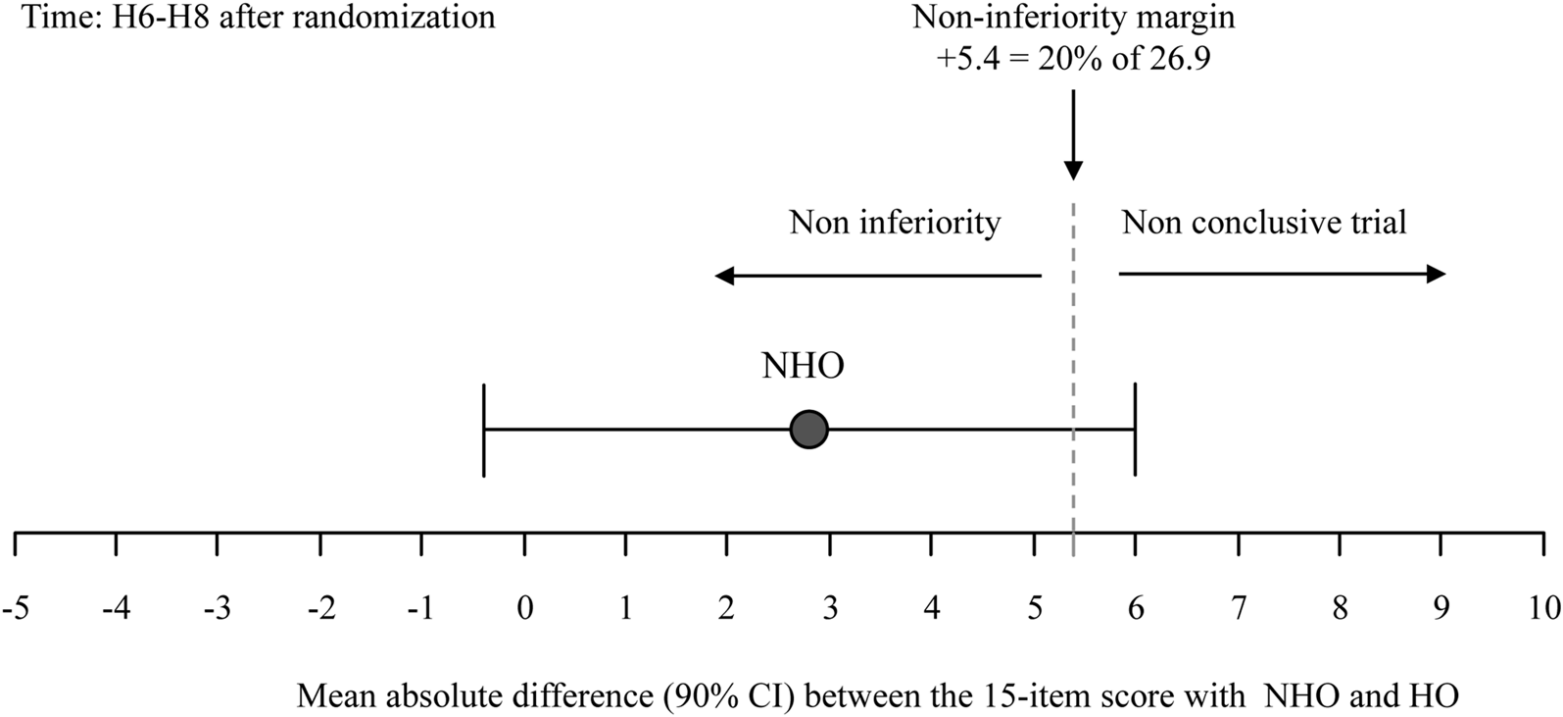
Absolute difference (with confidence interval) between the 15-item comfort scores at H6–H8 after randomization between humidified oxygen (HO) and non-humidified (NHO) oxygen. The non-inferiority margin was within the 90% CI of the absolute difference, meaning that the non-inferiority analysis was not conclusive

Figure 2 shows the results of the PP non-inferiority analysis for the 15-item discomfort score recorded at H6–H8. The mean value (± SD) of the 15-item discomfort score at H6–H8 was 26.9 ± 20.1 in the HO group (*n* = 136). The corresponding non-inferiority margin (Δ = 20% of 26.9) was calculated at +5.4. In the NHO group (*n* = 141), the mean value (± SD) of the 15-item comfort score was 29.7 ± 22.9. The absolute difference between HO and NHO comfort scores was 2.8 [90% CI − 0.4; +6.0]. The non-inferiority margin was within the 90% CI of the absolute difference, meaning that the non-inferiority analysis was not conclusive [13]. For sensitivity reasons, a mITT analysis was also performed [13]. It provided the same results as the PP analysis (not shown). Figure AF-3 illustrates the median (IQR) discomfort scores recorded in HO and NHO groups at H6–H8. In the HO group and in the NHO group, respectively, 17.6 and 19.0% of the patients (*p* = 0.77) had a 15-item comfort score of more than 45/150. Item-by-item results of the 15-item comfort score are provided in figure AF-4 of AF.

**Fig. 3:**
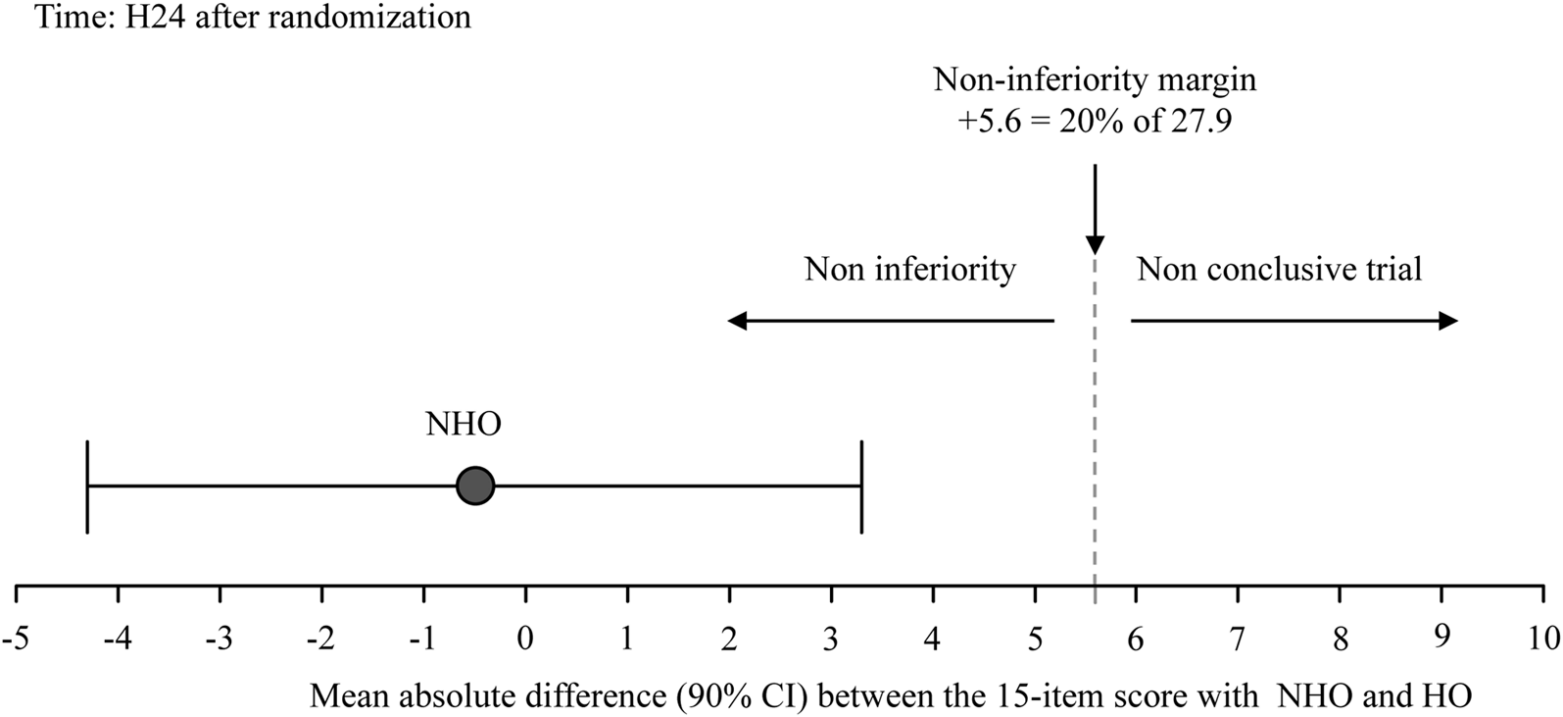
Absolute difference (with confidence interval) between the 15-item comfort scores at H24 after randomization between humidified oxygen (HO) and non-humidified (NHO) oxygen. NHO was non-inferior compared to HO in terms of comfort at H24

At H24, in PP analysis, the mean value (± SD) of the 15-item discomfort score was 27.9 ± 19.0 in the HO group (*n* = 72). The corresponding non-inferiority margin (Δ = 20% of 27.9) was calculated at +5.6. In the NHO group (*n* = 86), the mean value (± SD) of the 15-item comfort score was 27.4 ± 21.1. The absolute difference between HO and NHO comfort scores was − 0.5 [90% CI − 4.3; +3.3], meaning that NHO was not inferior to HO [13] (Fig. 3). The mITT analysis gave the same results (not shown). In the HO group and in the NHO group, respectively, 18.1 and 17.4% of the patients (*p* = 0.92) had a 15-item comfort score of more than 45/150. The median (IQR) comfort scores recorded in HO and NHO groups at H24 is displayed in Figure AF-3.

**Fig. 4:**
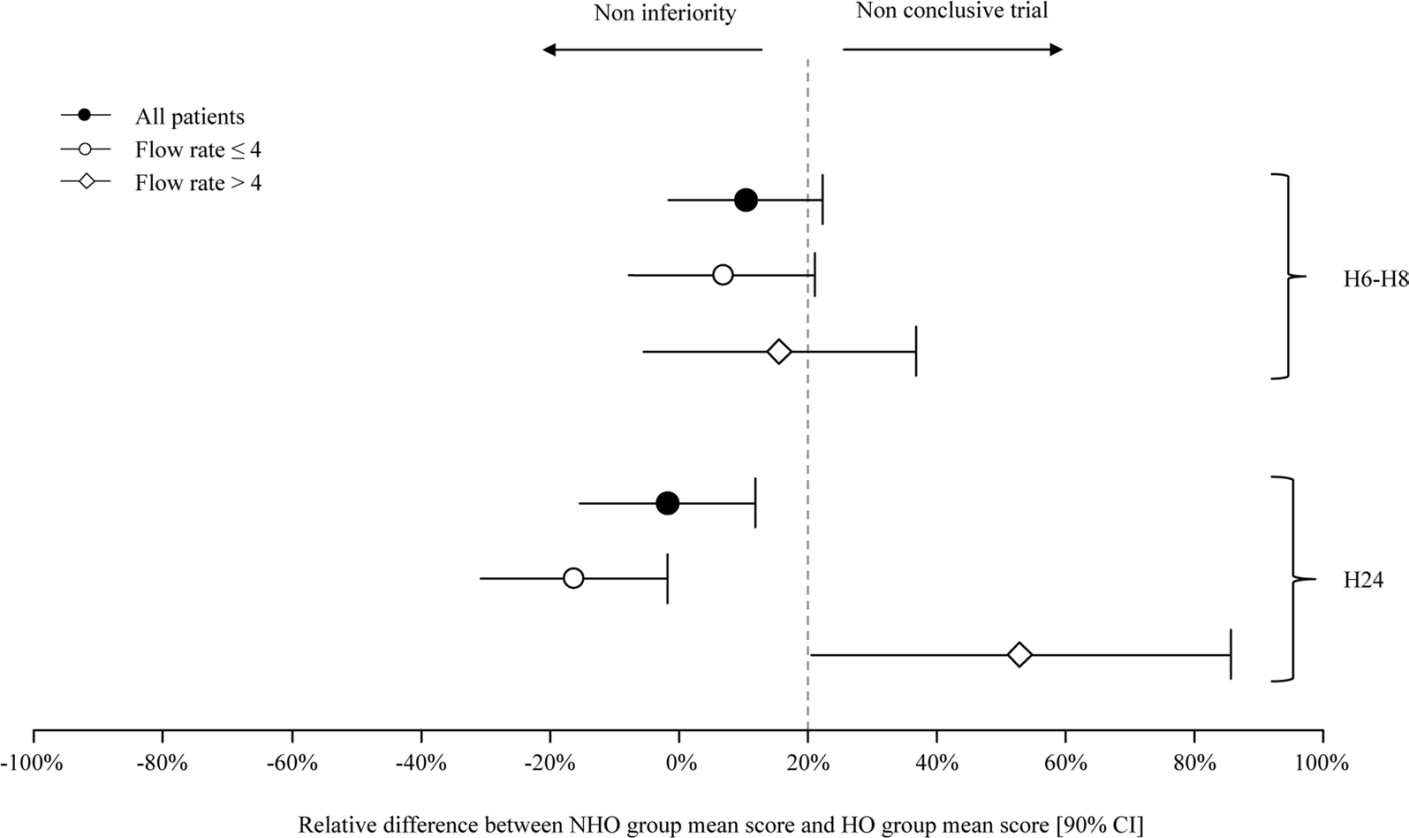
Relative difference (with confidence interval) between non-humidified oxygen (NHO) group mean score and humidified oxygen (HO) group mean score at H6–H8 and H24

The 15-item comfort scores recorded in the subgroups of patients who received oxygen, respectively, at flow rates ≤ 4 L/min and > 4 L/min were also compared for non-inferiority at H6–H8 and H24. Absolute and relative differences between NHO group and HO group mean scores are given in Table 2 for PP analysis. Relative differences are also displayed in Fig. 4. mITT gave the same results (not shown). As at H24 in the subgroup of patients receiving oxygen flow > 4 L/min, the confidence interval of the difference did not comprise either 0 or the non-inferiority margin, a post hoc inferiority analysis could be performed [13]. This analysis suggested inferiority of dry oxygen (*p* = 0.04) at H24 for oxygen flows > 4 L/min, meaning that discomfort was higher when no bubble humidification device was used.

**Table 2:** Effects of humidification strategy according to oxygen flow rate (non-inferiority analyses) at H6–H8 and H24 (per protocol analysis)

Oxygen flows	Results presentation	HO group comfort score	NHO group comfort score	Absolute difference between NHO group mean score and HO group mean score [90% CI]	Relative difference between NHO group mean score and HO group mean score [90% CI]	Interpretation
≤4 L/min H6–H8	Mean ± SD^a^	26.1 ± 19.4 20% NI margin =+5.2	27.9 ± 22.2	+ 1.8 [− 2.0; + 5.5]	+ 6.9 [− 7.7; + 21.1]	Non-conclusive
	Median [IQR]	21 [10.8; 37.5]	22 [11; 38.5]			
>4 L/min H6–H8	Mean ± SD^a^	29.3 ± 22.1 20% NI margin = + 5.9	33.9 ± 24.2	+ 4.6 [− 1.6; + 10.8]	+ 15.7 [− 5.5; + 36.9]	Non-conclusive
	Median [IQR]	22.5 [17.8; 36.5]	25 [18.5; 45]			
≤4 L/min H24	Mean ± SD^a^	29.6 ± 19.7 20% NI margin = + 5.9	24.8 ± 21.0	− 4.8 [− 9.1; − 0.5]	− 16.2 [− 30.7; − 1.7]	Non-inferiority
	Median [IQR]	25.5 [13.5; 42.8]	21 [12; 34]			
>4 L/min H24	Mean ± SD^a^	23.8 ± 16.6 20% NI margin = + 4.8	36.4 ± 19.5	+ 12.6 [+ 4.9; + 20.4]	+ 52.9 [+ 20.6; + 85.7]	Non-conclusive
	Median [IQR]	21 [12; 34]	33 [18.5; 43.5]			

*HO* humidified oxygen, *NHO* non-humidified oxygen, *CI* confidence interval

^a^For non-inferiority analysis

Bubble oxygen humidification had no effects on the need for intubation or noninvasive ventilation during ICU stay. The incidence of ear, nose or throat infection, the duration of ICU stay and ICU mortality were also similar in the HO and NHO groups. Detailed results are presented in AF (Table AF-2).

## Discussion

This randomized non-inferiority study demonstrated that oxygen therapy-related discomfort was relatively low among oxygen-dependent ICU patients. Conversely to the set hypothesis, this study did not allow to conclude that delivering dry oxygen was non-inferior than delivering oxygen moistened with bubble humidifiers after 6–8 h of oxygen administration. At H24, NHO was non-inferior to HO for the general patient. While NHO was demonstrated to be non-inferior to HO in the subgroup of patients receiving flows lower than or equal to 4 L/min of oxygen, in patients receiving more than 4 L/min of oxygen, a post hoc superiority analysis showed higher discomfort when dry oxygen was administered compared to bubble-humidified oxygen.

Both at H6–H8 and at H24, less than 20% of the patients reported significant discomfort scores (above 45/150). These score levels are much lower than previous data reported by Campbell et al. [2] who noticed that almost 50% of patients receiving oxygen at flow rates above 5 L/min either with or without humidification complained of significant discomfort. The discomfort recorded in the present study was also lower compared to the data of Chanques et al. [3] who reported that 29% of the patients reported moderate-to-severe discomfort for oxygen flows of more than 5 L/min. Possible explanations of these differences could be the use of non-comparable scales to assess comfort or different thresholds chosen to conclude to significant discomfort.

According to the previously published data [2, 3, 5, 12], it was anticipated to record, in our population, a mean 15-item discomfort score around 45/150 with a relatively low dispersion of the values. However, the mean comfort score values collected in our study were much lower than expected with very tight difference between the HO and NHO groups. In addition, the dispersion of the values was much broader than expected. Consequently, the power of our study turns out to be insufficient to conclude to non-inferiority of delivering dry oxygen. Considering the 15-item score values recorded in our study and a clinically significant threshold of 5.4 for the difference between comfort scores to conclude to non-inferiority, we found that one thousand four hundred and seventy-eight patients (739 patients per arm) would have been needed to have a power of 80% in this non-inferiority trial (absolute difference between HO and NHO comfort scores at H6–H8 of 2.8 points, SD = 20.1, α risk = 0.05, unilateral tests). This number seems definitely too high to repeat the study to be able to answer to the question of potential non-inferiority of using bubble humidifiers to deliver oxygen to ICU patients.

Compared to previously published studies focused on patients’ discomfort during oxygen therapy [2, 3, 5, 7, 12, 14–21], we used a 15-item specific comfort scale to assess all the components of oxygen therapy-related discomfort. We also evaluated comfort on a prolonged period of time.

In addition to the limited power of this study, some other limitations have to be mentioned. First, the discomfort scale used in the present study is new and not standardized. It was not validated in previous works. A scale validation was, however, performed in the same dataset but in other patient groups and is provided in AF. Second, no formal delirium diagnosis was performed before the oxygen-related discomfort assessment. However, only patients who had a Glasgow Coma Scale at 15/15 could be included in the study. To standardize the oxygen-related discomfort assessment, a health practitioner asked the patient to quote each of the fifteen items of the scale one by one. Third, as both comfortable and uncomfortable patients were included in the study, it could have been difficult to demonstrate a benefice potentially limited to the uncomfortable patients. Fourth, the subgroup analyses comprised a relatively small number of patients. Fifth, this study did not address the question of the impact of bubble humidification in tracheostomized or in pediatric patients. Finally, the conclusions of our study cannot be generalized for heated humidification, a technique with much higher potential to moisten delivered gas. Further studies are needed to explore the specific question of heated humidification on comfort at different gas flows.

## Conclusions

This study failed to demonstrate the non-inferiority, in terms of comfort, of dry oxygen compared to bubble-humidified oxygen in critically ill patients receiving oxygen after 6–8 h of oxygen therapy. No differences in clinical outcomes were found between dry oxygen and bubble-humidified oxygen. Subgroup analysis on data recorded after 24 h of oxygen therapy suggests that dry oxygen is non-inferior to bubble-humidified oxygen in patients receiving low (≤ 4 L/min) flow of oxygen but could be associated with a higher level of discomfort in patients receiving oxygen at higher (> 4 L/min) flows.

## Additional file

**Additional file 1.** The Oxyrea 15-item discomfort scale validation process and complementary data of the Oxyrea study.

## Abbreviations

AF: Additional files
CI: Confidence interval
ENT: Ear, nose or throat
H6–H8: 6th to 8th hour after inclusion
H24: 24th hour after inclusion
HO: Humidified oxygen
ICU: Intensive care unit
IQR: Interquartile range
NHO: Non-humidified oxygen
PP: Per protocol
mITT: Modified intention-to-treat
REVA network: Réseau européen de recherche en ventilation artificielle
SD: Standard deviation

## References

[1] Novaes MA, Knobel E, Bork AM, Pavão OF, Nogueira-Martins LA, Ferraz MB (1999). Stressors in ICU: perception of the patient, relatives and health care team. Intensive Care Med.

[2] Campbell EJ, Baker MD, Crites-Silver P (1988). Subjective effects of humidification of oxygen for delivery by nasal cannula. A prospective study. Chest..

[3] Chanques G, Constantin J-M, Sauter M, Jung B, Sebbane M, Verzilli D (2009). Discomfort associated with underhumidified high-flow oxygen therapy in critically ill patients. Intensive Care Med.

[4] Déry R, Pelletier J, Jacques A, Clavet M, Houde JJ (1967). Humidity in anaesthesiology. 3. Heat and moisture patterns in the respiratory tract during anaesthesia with the semi-closed system. Can Anaesth Soc J..

[5] Andres D, Thurston N, Brant R, Flemons W, Fofonoff D, Ruttimann A (1997). Randomized double-blind trial of the effects of humidified compared with nonhumidified low flow oxygen therapy on the symptoms of patients. Can Respir J.

[6] Wells RE, Perera RD, Kinney JM (1963). Humidification of oxygen during inhalational therapy. N Engl J Med.

[7] Estey W (1980). Subjective effects of dry versus humidified low flow oxygen. Respir Care..

[8] Wen Z, Wang W, Zhang H, Wu C, Ding J, Shen M (2017). Is humidified better than non-humidified low-flow oxygen therapy? A systematic review and meta-analysis. J Adv Nurs.

[9] Fulmer Snider (1984). ACCP-NHLBI National conference on Oxygen therapy. Arch Intern Med.

[10] Kallstrom TJ, American Association for Respiratory Care (AARC) (2002). AARC Clinical Practice Guideline: oxygen therapy for adults in the acute care facility–2002 revision & update. Respir Care.

[11] O’Driscoll BR, Howard LS, Earis J, Mak V (2017). British thoracic society emergency oxygen guideline group, BTS emergency oxygen guideline development group. BTS guideline for oxygen use in adults in healthcare and emergency settings. Thorax.

[12] Miyamoto K, Nishimura M (2008). Nasal dryness discomfort in individuals receiving dry oxygen via nasal cannula. Respir Care..

[13] Piaggio G, Elbourne DR, Pocock SJ, Evans SJW, Altman DG, CONSORT Group (2012). Reporting of noninferiority and equivalence randomized trials: extension of the CONSORT 2010 statement. JAMA.

[14] Roca O, Riera J, Torres F, Masclans JR (2010). High-flow oxygen therapy in acute respiratory failure. Respir Care..

[15] Sasaki H, Yamakage M, Iwasaki S, Mizuuchi M, Namiki A (2003). Design of oxygen delivery systems influences both effectiveness and comfort in adult volunteers. Can J Anesth..

[16] Eastwood GM, Reeves JH, Cowie BS (2004). Nasopharyngeal oxygen in adult intensive care - lower flows and increased comfort. Anaesth Intensive Care.

[17] Eastwood GM, Dennis MJ (2006). Nasopharyngeal oxygen (NPO) as a safe and comfortable alternative to face mask oxygen therapy. Aust Crit Care Off J Confed Aust Crit Care Nurses..

[18] Lee GJ, Lee SW, Oh Y-M, Lee JS, Lee S-D, Shin CS (2014). A pilot study comparing 2 oxygen delivery methods for patients’ comfort and administration of oxygen. Respir Care..

[19] Maggiore SM, Idone FA, Vaschetto R, Festa R, Cataldo A, Antonicelli F (2014). Nasal high-flow versus Venturi mask oxygen therapy after extubation. Effects on oxygenation, comfort, and clinical outcome. Am J Respir Crit Care Med.

[20] Lemiale V, Mokart D, Mayaux J, Lambert J, Rabbat A, Demoule A (2015). The effects of a 2-h trial of high-flow oxygen by nasal cannula versus Venturi mask in immunocompromised patients with hypoxemic acute respiratory failure: a multicenter randomized trial. Crit Care.

[21] Frat J-P, Thille AW, Mercat A, Girault C, Ragot S, Perbet S (2015). High-flow oxygen through nasal cannula in acute hypoxemic respiratory failure. N Engl J Med.

