# Supplementary material for: Effect on comfort of administering bubble-humidified or dry oxygen: the Oxyrea non-inferiority randomized study
Source: Ann Intensive Care. 2018 Dec 17;8:126. doi: 10.1186/s13613-018-0472-9 (PMC6297119; doi:10.1186/s13613-018-0472-9)
Supplement: Supplementary file 1 — Additional file 1. The Oxyrea 15-item discomfort scale validation process and complementary data of the Oxyrea study. [file 13613_2018_472_MOESM1_ESM.docx]

TITLE

Effect on comfort of administering bubble humidified or dry oxygen: the Oxyrea non-inferiority randomized study

AUTHORS

Laurent Poiroux* (corresponding author)

RN CCN

Medical intensive care department

Angers University Hospital, 4 rue Larrey 49933 Angers cedex France

[lapoiroux@chu-angers.fr](mailto:lapoiroux@chu-angers.fr)

Lise Piquilloud*

MD

Adult Intensive Care and Burn Unit

Lausanne University Hospital, rue du Bugnon 46, 1011 Lausanne, Switzerland

Medical intensive care department

[Lise.Piquilloud@chuv.ch](mailto:Lise.Piquilloud@chuv.ch)

Valérie Seegers

MD

Département de Biométrie

Institut de Cancérologie de l’Ouest, 15 avenue Bocquel, 49055 Angers cedex 02

[Valerie.Seegers@ico.unicancer.fr](mailto:Valerie.Seegers@ico.unicancer.fr)

Cyril Le Roy

RN

Medical Intensive care department

Angers University Hospital, 4, rue Larrey 49933 Angers cedex France

[Cyril.LeRoy@chu-angers.fr](mailto:Cyril.LeRoy@chu-angers.fr)

Karine Colonval

RN

Intensive care department

Orléans Regional Hospital, 4 avenue de l'hôpital 45067 Orléans cedex France

[karine.colonval@chr-orleans.fr](mailto:karine.colonval@chr-orleans.fr)

Carole Agasse

RN

Medical intensive care department

Nantes University Hospital, 1 place Alexis-Ricordeau 44093 Nantes Cedex 1 France

[Carole.AGASSE@chu-nantes.fr](mailto:Carole.AGASSE@chu-nantes.fr)

Vanessa Zinzoni

RN

Intensive care department

La Roche-sur-Yon Hospital, Boulevard Stéphane Moreau, 85925 La Roche-sur-Yon France

[vanessa.zinzoni@chd-vendee.fr](mailto:vanessa.zinzoni@chd-vendee.fr)

Vanessa Hodebert

RN

Intensive care unit

Saint-Malo Hospital, 1 Rue de la Marne, 35400 Saint-Malo France

[vanessa.hodebert@laposte.net](mailto:vanessa.hodebert@laposte.net)

Alexandre Cambonie

RN

Medical intensive care department

Poitiers University Hospital, 2 rue de la Milétrie 86000 Poitiers France

[alex.cambonie@wanadoo.fr](mailto:alex.cambonie@wanadoo.fr)

Josselin Saletes

Intensive care unit

RN

Le Mans Hospital, 194 avenue Rubillard 72037 Le Mans cedex 9 France

[josselinsaletes@gmail.com](mailto:josselinsaletes@gmail.com)

Irma Bourgeon

RN

Medical intensive care department

Henri Mondor University Hospital

51 avenue du Maréchal de Lattre de Tassigny, 94010 Créteil France

[irma.bourgeon-ghittori@aphp.fr](mailto:irma.bourgeon-ghittori@aphp.fr)

François Beloncle

MD

Intensive care department

Angers University Hospital 4, rue Larrey 49933 Angers cedex France

[Francois.Beloncle@chu-angers.fr](mailto:Francois.Beloncle@chu-angers.fr)

Alain Mercat

MD PhD

Intensive care department

Angers University Hospital 4, rue Larrey 49933 Angers cedex France

[AlMercat@chu-angers.fr](mailto:AlMercat@chu-angers.fr)

and [for the REVA Network](http://www.atsjournals.org.frodon.univ-paris5.fr/author/For+The+REVA+Research+Network)

* These two authors equally contributed to the present work.

**ADDITIONAL FILES**

1. **Participating centers**

The participating centers and the number of patients included in each center are given in table AF-1.

| Participating centers | Inclusions (n) |
| --- | --- |
| University Hospital of Angers | 110 |
| Regional Hospital of Orléans | 83 |
| University Hospital of Nantes | 58 |
| Hospital of La Roche/Yon | 38 |
| Hospital of Saint-Malo | 37 |
| University Hospital of Poitiers | 14 |
| Hospital of Le Mans | 8 |
| University Hospital Henri Mondor | 6 |
| Foch Hospital | 2 |

Table AF-1: List of the participating centers and number of inclusions

1. **Oxyrea 15-item discomfort scale: description and validation process**

- **15-item discomfort score description**

Briefly, the 15-item discomfort scale consists of a combination of the following 15 items: oral dryness, oral burning sensation, speaking difficulty, thirst, throat dryness, sore throat and / or swallowing difficulty, sensation of coolness or warmth in airways, nasal dryness, greater need to blow the nose, particular smell sensation, eye discomfort, chest discomfort, headache, discomfort related to ambient noise and abnormal taste in the mouth. Each item was quoted by the subject upon request between 0 (no discomfort) and 10 (worst imaginable discomfort). The global score was obtained by adding the points given to each of the 15 items. The final score is thus comprised between 0 and 150. The scale is illustrated in Figure AF-1.


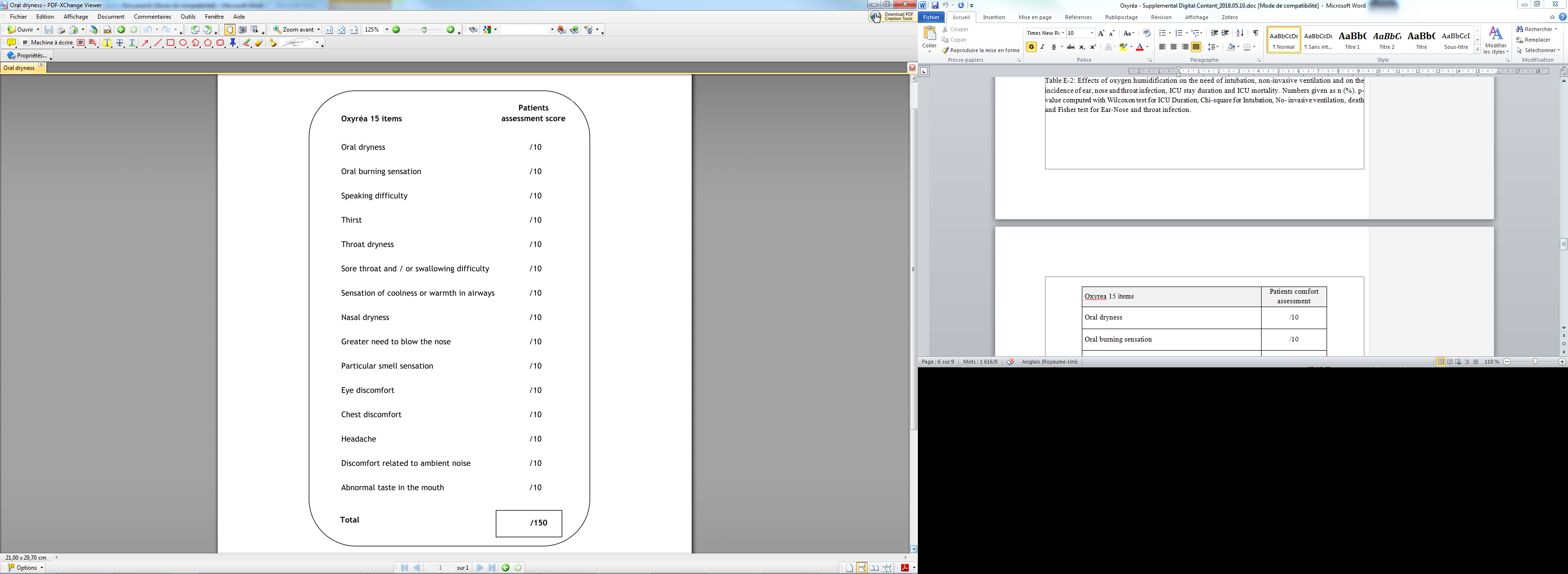


Figure AF-1: The 15-item discomfort scale

- **15-item discomfort score validation process: methodology**

In the present study, the 15-item scale was used to assess discomfort in 282 ICU subjects requiring oxygen therapy at 6 to 8 hours after oxygen therapy initiation. Overall, 278 discomfort evaluations were actually performed and are used for the present validation. To evaluate psychometrics properties of the scale, we defined learning and validation cohorts according to the interface used for oxygen therapy: nasal cannula (n=213) and facial mask (n=65) respectively. These learning and validation cohorts differ from the randomization group of the Oxyrea study. The unidimensionality of the scale was graphically assessed using scree plot [1]. According to Lance et al. recommendations [2], 100 simulated sets of similar size were generated and scree plots were measured to illustrate what “could be expected by chance”. The internal consistency was explored using inter-item correlation, item-total score correlation, Cronbach α coefficient (≥0.90 means excellent, ≥0.80 means good and ≥0.70 means acceptable [3], Split-half reliability (adjusted using the Spearman–Brown prophecy formula) and the composite reliability was computed using confirmatory factor analysis.

The missing data were not imputed.

- **15-item discomfort score validation process: results and results interpretation**

The number of missing answers was low in the learning cohort (6 missing values: one for “sore throat and/or swallowing difficulty”, four for “sensation of coolness or warmth in airways” and one for “eye discomfort”). There were no missing answers in the validation cohort. Figure AF-2 illustrates that, both in learning and validation cohorts, only one dimension emerged.

Figure AF-2: Scree plot items variables according respectively learning and validation cohorts

Table AF-2 presents values for internal consistency characterisation in learning and validation cohorts respectively: the Cronbach’s α coefficients were 0.857 (95%CI=0.820-0.896) and 0.875 (0.804-0.920). The Split half reliability adjusted using the Spearman Brown prophecy formula were 0.916 (0.889-0.936) and 0.930 (0.884-0.957). The Composite Reliability using confirmatory factor analysis were 0.867 and 0.847 respectively.

In other words, this results mean that the 15-item scale, used in the Oxyrea study, presents satisfying psychometric properties with a good internal consistency, with good Cronbach’s α and composite reliability and very good split-half reliability. As the scree plot demonstrated the unidimensionality of the 15-item score, there was no reasons to suppress any items. This work validates our 15-item scale, underlying its potential to assess the different aspects of oxygen therapy related discomfort.

|  | Learning cohort  (Nasal cannula) | Validation cohort  (Mask) |
| --- | --- | --- |
| Nb | 207 | 65 |
| Mean ± SD of inter-item correlation | 0.251 ± 0.061 | 0.292 ± 0.032 |
| Mean ± SD of total-item correlation | 0.544 ± 0.132 | 0.581 (0.057) |
| Cronbach’s α (95%CI) | 0.857 (0.820-0.896) | 0.875 (0.804-0.920) |
| Split-half reliability adjusted using the Spearman Brown prophecy formula | 0.916 (0.889-0.936) | 0.930 (0.884-0.957) |
| Composite Reliability using confirmatory factor analysis | 0.867 | 0.847 |

NB. The 95%CI of Cronbach was calculated using bootstrap adjusted quantiles.

Table AF-2: Internal consistency criteria to assess the 15-item scale

1. **Statistical software**

For all primary and secondary outcomes analyses the standard R package was used: R Core Team (2018). R: A language and environment for statistical computing. R Foundation for Statistical Computing, Vienna, Austria. URL [https://www.R-project.org/](https://www.r-project.org/).)

For the scale validation, the 2 following additional packages were used:

- Various procedures used in psychometry. R package version 1.1. [https://CRAN.R-project.org/package=psy](https://cran.r-project.org/package=psy))
- Lavaan: An R Package for Structural Equation Modeling. Journal of Statistical Software, 48(2), 1-36. URL <http://www.jstatsoft.org/v48/i02/>.)

1. **Additional detailed results**

Median discomfort score (box-plots) in the HO and NHO groups are illustrated in Figure AF-3. Effects of oxygen humidification on the need of intubation, non-invasive ventilation and on the incidence of ear, nose and throat infection, ICU stay duration and ICU mortality are mentioned in Table AF-3. Item by item results of the 15-item discomfort score are provided in Figure AF-4.


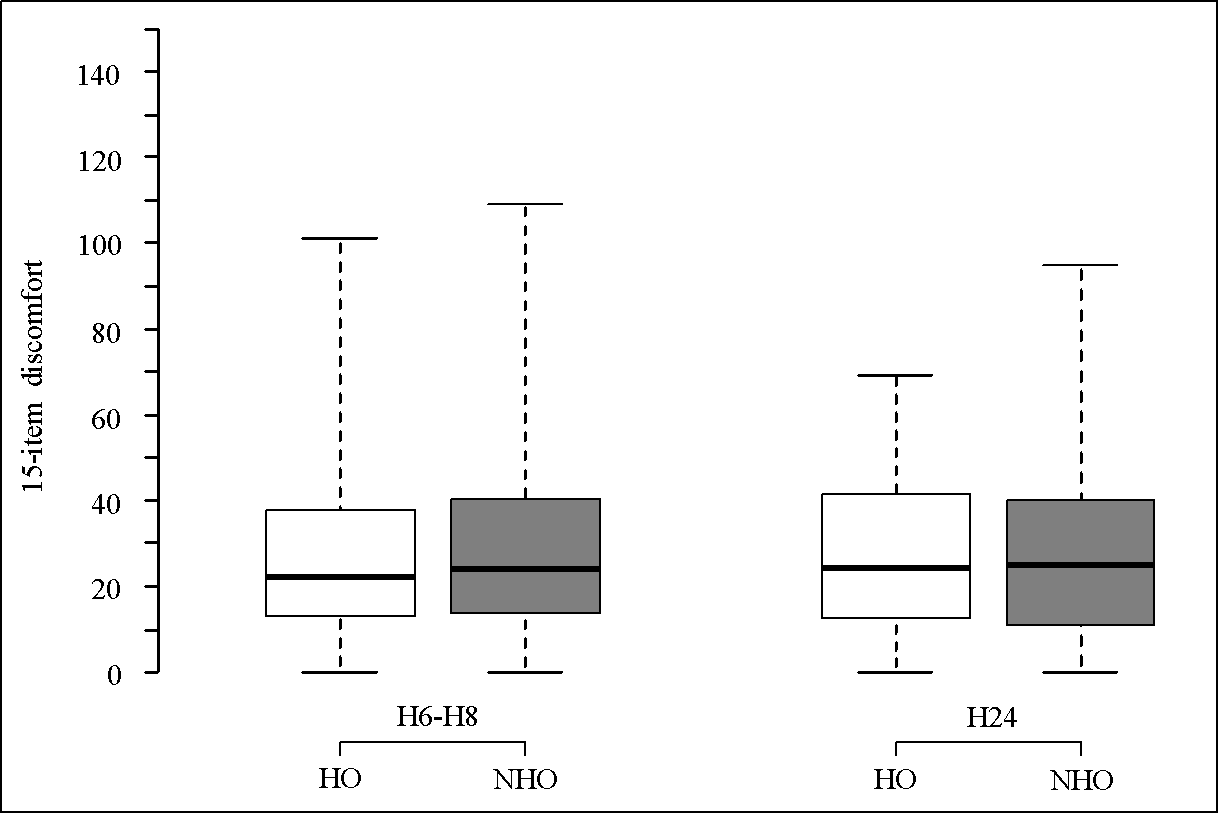


Figure AF-3: Fifteen-item discomfort scores at 6 to 8 hours and at 24 hours after inclusion for the group receiving humidified oxygen (HO) and for the group receiving non-humidified oxygen (NHO). In the box plots, the horizontal lines represent median values. The lower and upper parts of the boxes correspond to the 25th and 75th percentiles. Minimal and maximal values are also displayed.

Oral dryness

Oral burning sensation

Speaking difficulty

Thirst

Throat dryness

Coolness or warmth sensation

Swallowing difficulty

Need to blow

Nasal dryness

Particular smell

Eye

discomfort

Chest

discomfort

Headache

10

8

9

6

7

5

15-item comfort score

4

3

2

1

0

Noise

Abnormal taste

OH

ONH

OH

ONH

OH

ONH

OH

ONH

OH

ONH

OH

ONH

OH

ONH

OH

ONH

OH

ONH

OH

ONH

OH

ONH

OH

ONH

OH

ONH

OH

ONH

OH

ONH

Figure AF-4: The fifteen individual items of the 15-item discomfort scores at H6-H8 after inclusion for the humidified oxygen (HO) and the non-humidified oxygen (NHO) groups. In the box plots, the horizontal black lines represent median values. The lower and upper parts of the boxes correspond to the 25th and 75th percentiles. Minimal and maximal values are also displayed.

|  | Humidified oxygen  Therapy  (n=136) | Non-humidified oxygen  Therapy  (n=142) | p |
| --- | --- | --- | --- |
| Bronchoscopy n (%) | 7 (5.1%) | 8 (5.6%) | 0.85 |
| Intubation n (%) | 17 (12.5%) | 14 (9.9%) | 0.50 |
| Non-invasive ventilation n (%) | 9 (6.6%) | 7 (5.0%) | 0.55 |
| ENT infection n (%) | 1 (0.7%) | 0 | 0.49 |
| ICU stay duration (days) | 3 [1.0-6.0] | 3 [2.0-4.5] | 0.90 |
| Death n (%) | 8 (5.9%) | 7 (5.0%) | 0.74 |

Table AF-3: Effects of oxygen humidification on the need of bronchoscopy, intubation, non-invasive ventilation and on the incidence of ear, nose and throat infection, ICU stay duration and ICU mortality. Numbers given as n (%).

1. **References**

1. Cattell RB. The scree test for the number of factors. Multivariate behavioral research. 1966;1:245–276.

2. Lance CE, Butts MM, Michels LC. The sources of four commonly reported cutoff criteria: What did they really say? Organizational research methods. 2006;9:202–220.

3. Kline P. Handbook of psychological testing. Routledge; 2013.
